# Coronary conductance in the normal development of sheep during the perinatal period

**DOI:** 10.14814/phy2.15523

**Published:** 2022-12-02

**Authors:** Matthew W. Hagen, Samantha Louey, Sarah M. Alaniz, Laura Brown, Jonathan R. Lindner, Sonnet S. Jonker

**Affiliations:** ^1^ Center for Developmental Health Oregon Health & Science University Portland Oregon USA; ^2^ Knight Cardiovascular Institute, Oregon Health & Science University Portland Oregon USA; ^3^ Department of Pediatrics Perinatal Research Center University of Colorado Anschutz Medical Campus Aurora Colorado USA

**Keywords:** birth, coronary reserve, myocardial perfusion, parturition

## Abstract

Birth is associated with substantial shifts in cardiovascular physiology. Little is known about coronary vascular adaptations during this period. We used fetal and neonatal lambs to measure coronary function at late gestation (92% of term) and shortly after birth (5–6 days postnatal age). In each animal we measured unanesthetized myocardial perfusion and oxygen delivery using a circumflex artery flow probe. We used inflatable occluders and adenosine to determine coronary conductance and flow reserve. In a subset of animals, we used myocardial contrast echocardiography (MCE, anesthetized) to assess its utility as a tool for studying changes in regional myocardial perfusion in normal development. Separate age‐matched animals were instrumented with aortic and coronary sinus sampling catheters to determine myocardial oxygen extraction (unanesthetized). With an average of 17 days of developmental time separating our neonatal and fetal cohorts we found that heart‐to‐body weight ratio was significantly greater in neonates than fetuses. In resting animals, we found significant decreases in weight‐normalized perfusion of, and oxygen delivery to, neonatal relative to fetal myocardium. Similar results were seen when measuring baseline MCE‐derived perfusion. Pressure‐flow relationship studies revealed lower baseline and maximal coronary conductance in neonates than fetuses, with similar coronary flow reserve between groups. There was greater oxygen extraction in neonates than fetuses. Combined analysis of oxygen extraction with coronary flow suggested greater oxygen consumption by the fetal than neonatal myocardium. We conclude that, during the immediate perinatal period, cardiac growth outpaces coronary microvascular growth resulting in lower capacity for microvascular perfusion in the early neonate.

## Graphical abstract

We characterize coronary perfusion, conductance, and reserve in late gestation fetal and early neonatal lambs to address the dearth of knowledge about coronary adaptations to the birth transition in normal development. Using implanted flow probes and myocardial contrast echocardiography, we find that resting perfusion, flow capacity, and oxygen delivery to and consumption by the neonatal myocardium are all lower than by fetal myocardium, while coronary flow reserve is unchanged.

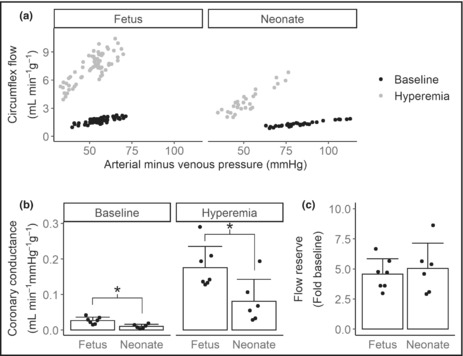

## 1 INTRODUCTION

The birth transition from fetal to neonatal life is a near‐universal mammalian experience, and is accompanied by a substantial change in cardiovascular physiology. Systemic vascular resistance increases with the loss of placental circulation, arterial oxygen content increases with ventilation of the lungs, and systemic metabolic demand increases (Morton & Brodsky, 2016; Singer & Mühlfeld, 2007). Birth also coincides with important changes in cardiac growth patterns in large mammals, with the shift from hyperplastic to hypertrophic cardiomyocyte growth occurring near parturition (Adler & Costabel, 1975; Austin et al., 1995; Burrell et al., 2003; Jonker et al., 2015). We know that the capacity for adaptive myocardial angiogenesis decreases with advancing postnatal age (Flanagan et al., 1991, 1994), and that endothelial cells of existing vessels are limited in their contribution to neonatal coronary angiogenesis (Lu et al., 2021; Tian et al., 2014). However, to date no direct investigation of the changes in coronary function in response to normal birth has been reported.

Most investigations of the growth and function of the coronary vascular system in normal development have focused either on characterizing capillary density across postnatal life (Roberts & Wearn, 1941), or defining coronary flow capacity across a broad timeline from fetus through adulthood (Fisher et al., 1982). More recent work has defined changes to coronary vascular function in response to deviations from normal development (Jonker et al., 2016, 2020). To test our hypothesis that the birth transition would coincide with increases in myocardial oxygen consumption and flow reserve, we studied late gestation sheep fetuses (135–136 days gestational age [dGA]; term is 147 days) and early postnatal lambs (5–6 days old). In order to define the changes occurring in the coronary vasculature during a narrow developmental window surrounding birth, we measured myocardial perfusion, coronary conductance, and coronary flow reserve using transit time flow probes which are well established tools for the study of fetal coronary function (Davis et al., 2003; Giraud et al., 2006; Jonker et al., 2020; Karamlou et al., 2019). We also used myocardial contrast echocardiography (MCE) with a subset of animals in order to understand the potential utility of this less invasive technique for the study of regional microvascular function in normal development. Finally, in a separate cohort of animals we measured myocardial oxygen extraction in order to begin to understand how changes in oxygen availability affect the observed changes in coronary flow.

## 2 METHODS

### 2.1 Ethics statement

Our animal experiments were approved by the Institutional Animal Care and Use Committee of Oregon Health & Science University, which is accredited by AAALAC International.

### 2.2 Animal groups

**FIGURE 1:**
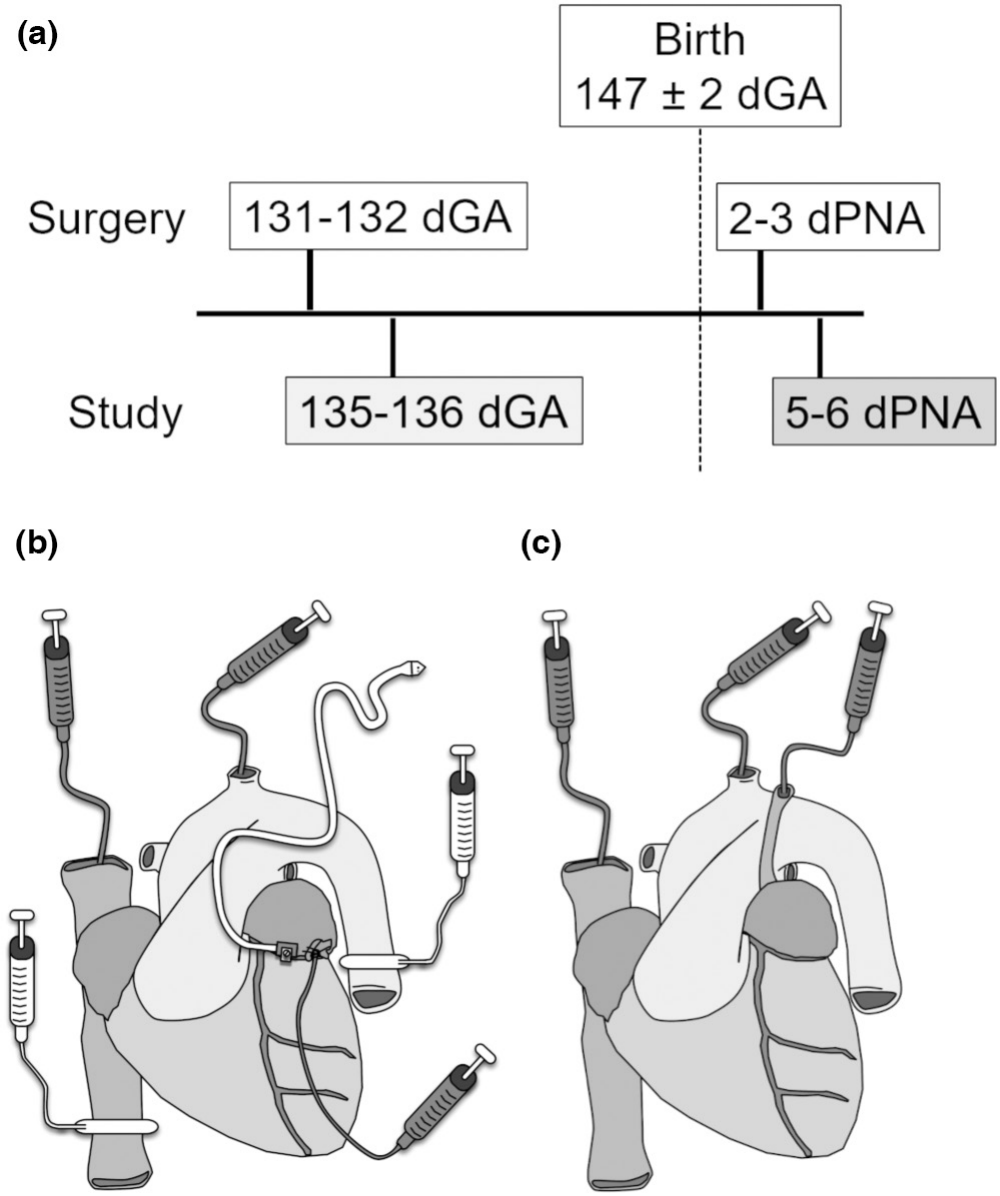
Study and instrumentation schematics. (a) Study timeline. Fetuses were instrumented at 131 or 132 dGA and terminal study took place at 135 or 136 dGA. Lambs were born at 147 ± 2 dGA, instrumented at 2 or 3 dPNA and studied at 5 or 6 dPNA. (b) Coronary flow cohort instrumentation for fetuses and lambs. Clockwise from top left: Superior vena cava catheters (via jugular vein), aortic arch catheters (via carotid artery), circumflex artery flow probe, postductal aorta occluder, left atrial catheter, inferior vena cava occluder. (c) Oxygen extraction cohort instrumentation for lambs. Clockwise from top left: Superior vena cava catheter (via jugular vein), aortic catheter (via carotid artery), coronary sinus catheter (via hemiazygos vein). In (b) and (c), gray syringes denote fluid‐filled catheters, white syringes denote air‐inflatable vascular occluders, and the white cap denotes the signal cable from the flow probe.

Four groups of animals were studied: fetuses and neonates instrumented for measurement of coronary hemodynamics (coronary flow cohorts) and fetuses and neonates instrumented for determination of myocardial oxygen extraction (oxygen extraction cohorts). Instrumented lambs, and ewes carrying instrumented fetuses, were euthanized at study endpoints, as described below. Ewes that delivered lambs but which never underwent surgery themselves were returned to our vendor following conclusion of the study. Experimental timelines are illustrated in Figure 1a.

#### 2.2.1 Coronary flow cohorts

Eight singleton fetuses were instrumented for the fetal coronary flow cohort. One fetus died from surgical complications in the 24 h following instrumentation, leaving 7 (2 female, 5 male) for study by transit‐time flow probes. Of these, 4 (1 female, 3 male) were also studied with MCE.

Ten lambs from singleton pregnancies were instrumented for the neonatal coronary flow cohort. One lamb was euthanized due to surgical complications. One lamb was euthanized the day before the planned endpoint due to flow probe failure. The remaining lambs survived to the study endpoint, however 2 were excluded from analysis: one for persistent postoperative acidosis and one for an unventilated lung lobe and pericardial adhesions found on necropsy. This left 6 lambs (2 male, 4 female) studied by transit‐time flow probe. Of these, 3 (1 male, 2 female) were also studied with MCE.

#### 2.2.2 Oxygen extraction cohorts

Fetal oxygen extraction data were taken from a previously published study (Jonker et al., 2020), and included 7 fetuses (2 females from twin pregnancies, 4 males from twin pregnancies, 1 male from a singleton pregnancy). Techniques for measurement of oxygen extraction (described below) were identical between this earlier work and that reported here.

Five male lambs from twin pregnancies were instrumented for the neonatal oxygen extraction cohort. All survived to the study endpoint; however, data could not be collected from one because of a clotted coronary sinus catheter.

### 2.3 Surgical instrumentation

Time‐bred pregnant ewes of mixed western breed were obtained from local vendors. Common elements of surgical instrumentation in the fetal and neonatal cohorts are illustrated in Figure 1b (coronary flow cohorts) and Figure 1c (oxygen extraction cohorts).

#### 2.3.1 Fetal instrumentation

For the coronary flow cohort, singleton fetuses of both sexes were instrumented at 131 or 132 dGA. Maternal anesthesia was induced with ketamine (400 mg, intravenous [iv]) and diazepam (10 mg, iv). Ewes were intubated, and mechanically ventilated with a mix of oxygen (2 L min^−1^), nitrous oxide (0.7 L min^−1^), and isoflurane (1.5%–2%). The uterus was exposed via abdominal midline incision and a hysterotomy was performed. Two saline‐filled vinyl catheters were placed in one carotid artery (0.86 × 1.32 mm inner diameter (ID) × outer diameter (OD), 1.19 × 1.80 mm ID×OD, tips advanced to the aortic arch, Scientific Commodities, Lake Havasu City, AZ, USA). Another two saline‐filled vinyl catheters were placed in one jugular vein (both 1.19 × 1.80 mm ID×OD, tips advanced to the superior vena cava). The thoracic cavity was accessed by left thoracotomy. Deflated occluders made of silicone elastomer (10–12 mm, In Vivo Metric, Healdsburg, CA, USA) were placed around the inferior vena cava and descending postductal aorta. One saline‐filled vinyl catheter (0.86 × 1.32 mm ID×OD with a 1.80 mm OD cuff over the inserted end) was placed in the left atrium via direct puncture. A 3 mm transit‐time flow probe (Transonic, Ithaca, NY, USA) was placed around the circumflex artery just below the left atrial appendage. Catheters and cables were anchored to the fetal skin, along with a vinyl amniotic sac catheter (1.19 × 1.80 mm ID×OD). Free ends were tunneled subcutaneously and exteriorized to a pouch sutured on the flank of the ewe. Fetal and maternal incisions were closed in layers, and ciprofloxacin (2 mg) and penicillin G (1 × 10^6^ U) were delivered into the amniotic sac. Ewes were returned to housing pens after weaning from anesthesia and when spontaneously breathing, extubated once swallowing, and continuously monitored until standing and eating, at which point buprenorphine (0.3 mg, subcutaneous [sc]) and slow release buprenorphine (0.05 mg kg^−1^, sc) were given. Food and water were available ad libitum once the ewe was recovered.

#### 2.3.2 Neonatal instrumentation

For the coronary flow cohort, lambs of either sex from singleton pregnancies were born by spontaneous vaginal delivery; surgeries were performed at 2 or 3 days postnatal age (dPNA). Lambs were pretreated with carprofen (2 mg kg^−1^ sc), and anesthesia was induced with ketamine (3 mg kg^−1^, iv) and diazepam (0.5 mg kg^−1^ iv). Lambs were intubated and mechanically ventilated with oxygen (2 L min^−1^) and isoflurane (1.5%–2%). Lambs were instrumented with vascular catheters, an atrial catheter, vascular occluders, and a circumflex artery flow probe as described for fetuses, above.

For the separate oxygen extraction cohort, surgical induction, incisions, and recovery were the same as for the coronary flow cohort. Oxygen extraction cohort lambs were instrumented with one saline‐filled catheter each in a carotid artery (1.19 × 1.80 mm ID×OD, tip advanced to the aortic arch) and jugular vein (1.19 × 1.80 mm ID×OD, tip advanced to the superior vena cava), and a saline‐filled catheter in the hemiazygos vein (0.86 × 1.32 mm ID×OD with a 3 cm vented silastic cuff 2.16 mm OD, tip advanced to the coronary sinus).

For all lambs, catheters were tunneled subcutaneously and exteriorized to the back, secured using body netting, and protected with a jacket. Incisions were closed in layers, and air evacuated from the thoracic cavity using a water‐sealed chest tube and manual positive pressure ventilation. They were recovered in the operating room until awake. Following extubation but prior to wakefulness, lambs were given corn syrup on their gums and at least 100 ml expressed sheep milk via gastric tube. They were continuously monitored until ambulatory and feeding from the ewe. Lambs were given a second dose of carprofen (2 mg kg^−1^ sc) 24‐h after pre‐dosing.

### 2.4 Resting hemodynamic study

#### 2.4.1 Fetal hemodynamics

Two days following surgical recovery, ewes were moved to stanchions which permitted standing or lying down at will and access to food and water ad libitum. Very low‐flow heparinized lactated Ringer's solution was infused into all fetal catheters (Miniplus 3, Gilson, Middleton, WI, USA) to maintain patency for continuous pressure recording. In‐line transducers (Transpac IV, Abbott, Abbott Park, IL, USA) were calibrated against a mercury manometer, and connected to a bridge amplifier and recorder (PowerLab, ADInstruments, Colorado Springs, CO, USA) from which continuous hemodynamic data were recorded. Pressures were corrected daily for transducer voltage drift. Vascular pressures were normalized to intra‐amniotic pressure. Heart rate was determined from the arterial pressure waveform. The flow probe was connected to a flow meter (Transonic). All data were continuously recorded at 40 Hz. Daily hemodynamics were determined by averaging approximately 2 h of recorded data collected each morning before the arrival of study or animal care staff. Arterial blood samples were collected at the end of this recording period and were analyzed immediately. The partial pressures of oxygen and carbon dioxide, hemoglobin concentration, oxygen content, and pH were measured using a blood gas analyzer (ABL 825, Radiometer America, Cleveland, OH, USA; temperature adjusted to 39°C); hematocrit was measured by capillary centrifugation.

#### 2.4.2 Neonatal hemodynamics

On 2 consecutive days beginning at 4 or 5 dPNA, neonates in the coronary flow cohort were placed in slings, in which they had been trained daily since birth to rest quietly. Vascular catheters and the flow probe were connected to transducers and a flow meter as described above. Thirty minutes of resting hemodynamics were recorded daily, after which an arterial blood sample was collected and analyzed as described above.

### 2.5 Adenosine dose–response

When fetuses were 134 or 135 dGA and lambs were 4 or 5 dPNA, an adenosine dose–response experiment was performed to identify the infusion rate which would induce maximum coronary vasodilation with minimal effect on arterial pressure. Fetuses (body weight estimated at time of surgery) and neonates were given atropine (0.5 mg kg^−1^) and propranolol (1 mg kg^−1^) intravenously (bolus) to control heart rate. Adenosine was infused into the left atrium at 4 to 8 different increasing rates ranging from 4 to 600 μg min^−1^ kg^−1^ to induce coronary vasodilation. Systemic pressures and circumflex flow were monitored in real time. Experiments concluded when the plateau phase of the sigmoidal dose–response relationship was observed.

### 2.6 Pressure‐flow relationships

When fetuses were 135 or 136 dGA and lambs were 5 or 6 dPNA, pressure‐flow relationship studies were performed to determine baseline and hyperemic coronary conductance and coronary flow reserve. Fetuses and lambs were given atropine and propranolol as above, and the left atrial catheter was pre‐filled with adenosine. A pressure‐flow relationship was determined by inflating either the inferior vena cava or post ductal aortic occluder with air over several seconds (inflation order was randomized between animals). This was repeated during adenosine‐mediated hyperemia using the adenosine rate determined from the dose–response study on the previous day (159 ± 68 μg min^−1^ kg^−1^). Each series of inflations was performed in at least triplicate. Example pressure‐flow data are shown in Figure 3a.

### 2.7 Myocardial imaging studies (terminal)

A subset of animals in the coronary flow cohorts underwent an MCE study following the pressure‐flow study.

#### 2.7.1 Fetal MCE preparation

Ewes were anesthetized as described above and ventilated with oxygen (2 L min^−1^) and isoflurane (1.5–2%). The maternal abdominal incision was re‐opened and extended into a Mercedes incision to avoid compression of the umbilical cord. The fetus was exteriorized through a hysterotomy and exposed to the level of the diaphragm.

#### 2.7.2 Neonatal MCE preparation

Neonatal anesthesia was induced as described above, and lambs were mechanically ventilated with oxygen (2 L min^−1^) and isoflurane (1.5%–2%).

#### 2.7.3 Myocardial contrast echocardiography (fetuses and lambs)

Fetal and neonatal hemodynamics were recorded throughout the echocardiography study, as described above, albeit under anesthesia. Arterial blood samples were collected immediately before imaging. Atropine and propranolol were given immediately before imaging as described above.

MCE was performed using a phased array transducer at 1.5 MHz (4V1c‐s, Sequoia 512, Siemens Medical Systems, Malvern, PA, USA) to determine microvascular perfusion in the left ventricular free wall myocardium, similar to a previous study (Jonker et al., 2016). The non‐linear signal component for microbubbles was detected using multi‐pulse phase‐and amplitude‐modulation at a mechanical index of 0.18 and a dynamic range of 55 dB. Gain settings were optimized and held constant per animal. One vial of microbubble ultrasound contrast agent (DEFINITY, Lantheus Medical Imaging, North Billerica, MA) was diluted in 15 ml saline and infused systemically via the superior vena cava. Blood pool (*I*_*B*_) imaging of the left ventricular cavity was performed with an infusion rate of 30 ml h^−1^. When needed for visualization of myocardial perfusion, infusion was increased to 60 ml h^−1^. Perfusion imaging was performed at end systole. Images were acquired for a minimum of 8 s after a high‐power (mechanical index 1.8) five‐frame destructive pulse sequence.

For MCE analysis, signal from non‐capillary vessels was eliminated by background subtraction of the first end‐systolic frame following destruction from subsequent frames. Time‐intensity data after the destructive pulse sequence were fitted to the function:

$$y=A\left(1-e^{-\mathrm{\beta t}}\right)$$

where *y* is intensity at time *t*, *A* is the plateau intensity reflecting relative microvascular blood volume, and the rate constant *β* represents the microvascular flux rate (s^−1^). Absolute microvascular blood volume (ABV; ml g^−1^) was quantified by scaled comparison of *A* with *I*_*B*_:

ABV=A1.06×IB×F

where *I*_*B*_ is the blood volume intensity, 1.06 is tissue density (g cm^−1^) and *F* is the scaling factor to correct for differences in infusion rates. Absolute microvascular blood flow (MBF; ml min^−1^ g^−1^) is the product of ABV and *β*, multiplied by 60 s min^−1^.

### 2.8 Myocardial oxygen consumption study

At 4 and 5 dPNA, neonates in the oxygen extraction cohort had arterial and coronary sinus blood samples simultaneously collected. Oxygen content was determined as described above. This was the only study completed with these animals.

### 2.9 Euthanasia

Fetuses and neonates were systemically anticoagulated by bolus delivery of heparin (6000 U, iv) immediately prior to euthanasia. Euthanasia of lambs and ewes carrying instrumented fetuses was carried out by giving an overdose of a commercial barbiturate (iv, SomnaSol Covetrus, Dublin, OH, USA). Coronary flow cohort animals were euthanized within 4 h of pressure‐flow study, and oxygen extraction cohort lambs were euthanized within an hour of 5 dPNA sampling. MCE animals were euthanized immediately after imaging, while anesthetized. Hearts of anesthetized fetuses were arrested in diastole by bolus delivery of saturated potassium chloride (iv).

### 2.10 Evans blue procedure

Immediately following euthanasia, the hearts from the coronary flow cohort were processed as described (Jonker et al., 2020). Following excision and trimming, the flow probe was removed leaving the circumflex artery intact. Hearts were trimmed in a standardized manner, and weighed whole. The left main coronary ostium was cannulated and the cannula advanced through the circumflex branch to the level of the flow probe. Saline saturated with Evans blue dye was slowly infused until dye was seen emerging from the coronary sinus. The myocardium was dissected into 5 pieces: left atrium (including atrial septum), right atrium, left ventricular free wall, ventricular septum, right ventricular free wall. Each structure was further dissected to separate the stained from unstained tissues. The total mass of myocardium stained blue was the area served by the arterial segment measured with the flow probe, and was used to derive flow per mass.

### 2.11 Explanation of parameters

*Arterial minus venous pressure* (mmHg) is the difference between mean systemic arterial and venous pressures.

*Cardiac rate pressure product* (RPP, mmHg min^−1^) is the product of arterial minus venous pressure and heart rate and is our estimate of cardiac work.

*Circumflex flow* (ml min^−1^ g^−1^) is flow through the circumflex artery normalized to the weight of myocardium stained during the Evan's blue procedure described above.

*Conductance* (ml min^−1^ mmHg^−1^ g^−1^ **)** is the relationship between coronary flow and arterial minus venous pressure, calculated here as the slope of each pressure‐flow relationship.

*Flow reserve* is the degree by which coronary flow can be increased by vasodilation. It is expressed as fold difference and calculated by comparing the flows fitted to the baseline and hyperemic pressure‐flow relationships at each animal's resting daily arterial minus venous pressure.

*O*_*2*_ *extraction* (ml dl^−1^) is the difference between arterial and coronary sinus oxygen contents.

*Microvascular blood flow* (MBF, ml min^−1^ g^−1^) is the coronary microvascular flow per gram of myocardium as determined by MCE.

*Microvascular conductance* (ml min^−1^ mmHg^−1^ g^−1^) is the relationship between coronary microvascular flow and arterial minus venous pressure, calculated here as the quotient of MBF and arterial minus venous pressure.

*O*_*2*_ *delivery* (ml mmHg^−1^ g^−1^) is the oxygen brought to the myocardium by arterial blood normalized to RPP. It is calculated as the product of arterial oxygen content and either coronary flow or MBF.

### 2.12 Statistics

Results are reported as mean ± standard deviation (*SD*). All statistics were calculated using R (v4.1.1 R Foundation for Statistical Computing, Vienna, Austria) (R Core Team, 2022). All hypothesis tests concerned the differences between fetuses and neonates. One experimental unit was defined as one animal. Data determined by the Bartlett test to be normally distributed were computed using two‐sided unpaired Student's *t*‐tests; those determined to be non‐normally distributed were computed using the Mann–Whitney *U*‐test. Data were considered statistically significant when *p* < 0.05.

## 3 RESULTS

Fetuses were studied at 135 or 136 dGA. The gestational age at birth of the neonatal group was 147 ± 2 dGA; lambs were studied at 5 or 6 days after birth. On average, 17 developmental days separated the groups.

### 3.1 Morphometry

Morphometric data are reported for the coronary flow cohort animals. Lambs were 40% heavier than fetuses (Table 1). Heart weight was 72% greater in lambs than fetuses. Consequently, the heart to body weight ratio was 25% greater in lambs than fetuses. All chamber walls in the lamb heart were heavier than in the fetal heart; the neonatal left ventricle was twice as heavy as that of the fetus.

**TABLE 1:** Weights

	Fetus	Neonate	*p*‐value
N (male)	7 (5)	6 (2)	
Body (kg)	5.2 ± 0.6	7.2 ± 1.4	0.0172
Heart (g)	31.7 ± 5.3	54.5 ± 10.4	0.0017
Heart to body (g/kg)	6.1 ± 0.5	7.6 ± 0.4	<0.0001
Left atrium (g)	2.8 ± 0.7	4.9 ± 1.5	0.0154
Right atrium (g)	2.0 ± 0.4	3.4 ± 0.8	0.0068
Left ventricle (g)	8.4 ± 1.4	18.0 ± 3.4	0.0004
Right ventricle (g)	8.5 ± 1.8	12.9 ± 3.2	0.0200
Ventricular septum (g)	5.4 ± 1.2	10.0 ± 1.9	0.0007

*Note*: Sample sizes, sex distributions, and endpoint body, heart, and heart wall weights for fetuses (135 or 136 dGA) and neonates (5 or 6 dPNA) from the coronary flow cohort. Mean ± *SD*, differences assessed by *t*‐test.

**FIGURE 2:**
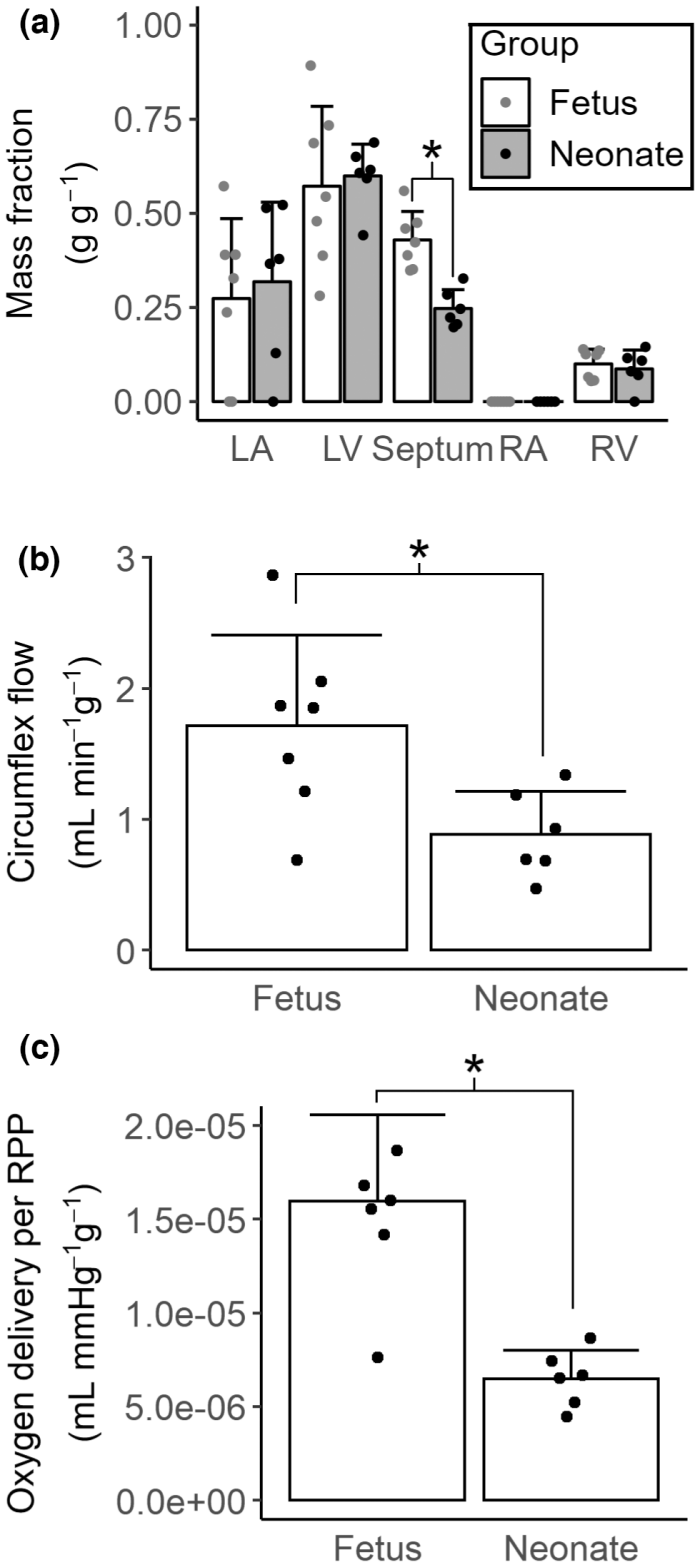
Resting (unanesthetized) coronary flow assessed in fetuses (135 or 136 dGA, *N* = 7) and lambs (5 or 6 dPNA, *N* = 6). (a) Mass fractions of major heart structures served by the circumflex artery segment measured by the flow probe (left atrium (LA), left ventricle (LV), septum, right atrium (RA), and right ventricle (RV)). Only flow to the septum was changed by age (*t*‐test, *p* < 0.001). (b) Circumflex flow per gram of myocardium (*t*‐test, *p* = 0.020). (c) Oxygen delivery per RPP per gram of myocardium. (Mann–Whitney *U*‐test, *p* = 0.002). Columns are means, error bars + *SD*, dots are individual data. *p < 0.05.

The circumflex artery at the level of the flow probe placement perfuses just over half of the left ventricular free wall, one quarter (in neonates) to just under a half (in fetuses) of the septum, less than a third of the left atrium, and about a tenth of the right ventricular free wall (Figure 2a). The circumflex perfuses a greater portion of the fetal than neonatal septum (42.9 ± 7.6% vs 24.7 ± 5.0%, *p* < 0.001), all other structures' perfusion is similar between age groups (Figure 2a).

### 3.2 Blood composition and characteristics (unanesthetized)

Unanesthetized study day arterial blood composition and characteristics are reported for the coronary flow cohort animals. Arterial pH was similar between fetuses and neonates (Table 2). The partial pressure of oxygen was 3.7‐fold greater in neonates than fetuses, while arterial blood oxygen content was 60% greater. Neonatal partial pressure of CO_2_ was 84% of the fetal value. Neonatal hematocrit was 80% that of the fetus. Hemoglobin content was not different between fetuses and neonates.

**TABLE 2:** Unanesthetized arterial blood parameters, and hemodynamics

	Fetus	Neonate	*p*‐value
pH	7.380 ± 0.030	7.360 ± 0.040	NS
PCO_2_ (mmHg)	49 ± 2	41 ± 4	0.002
PO_2_ (mmHg)	22 ± 4	83 ± 9	<0.0001
O_2_ content (mg/dl)	7.8 ± 2.0	12.7 ± 2.0	0.001
Hemoglobin (g/dl)	11.0 ± 2.0	10.1 ± 1.8	NS
Hematocrit (%)	35 ± 7	28 ± 5	0.075
Arterial—venous pressure (mmHg)	49.1 ± 3.9	78.1 ± 10.7	0.001
Heart rate (min^−1^)	161 ± 12	214 ± 24	0.002
RPP (mmHg min^−1^)	7926 ± 1076	16,724 ± 3089	0.001
Circumflex flow (ml min^−1^)	12.6 ± 4.2	14.1 ± 6.2	NS

*Note*: Measurements made in resting, unanesthetized animals from coronary flow cohorts without autonomic blockade in fetuses (135 or 136 dGA, *N* = 7) and neonates (5 or 6 dPNA, *N* = 6). RPP (rate pressure product). Mean ± *SD*. Nonparametrically distributed data analyzed by Mann–Whitney *U*‐test (arterial–venous pressure, RPP), all others analyzed by *t*‐test. *p* > 0.05 considered not statistically significant (NS).

### 3.3 Resting hemodynamics, coronary conductance, and reserve (unanesthetized)

Resting heart rate and arterial minus venous pressure were both significantly higher in neonates than fetuses (Table 2). Consequently, the neonatal rate pressure product (RPP) was twice as high as the fetal RPP. Resting weight‐normalized circumflex flow was half in neonates what it was in fetuses (Figure 2b, 0.88 ± 0.33 vs 1.72 ± 0.69 ml min^−1^ g^−1^, *p* = 0.020). Weight‐normalized oxygen delivery per RPP was less than half in the neonate what it was in the fetus (Figure 2c, 6.5 × 10^−6^ ± 1.5 × 10^−6^ vs 1.6 × 10^−5^ ± 4.6 × 10^−6^ ml mmHg^−1^ g^−1^, *p* = 0.002).

**FIGURE 3:**
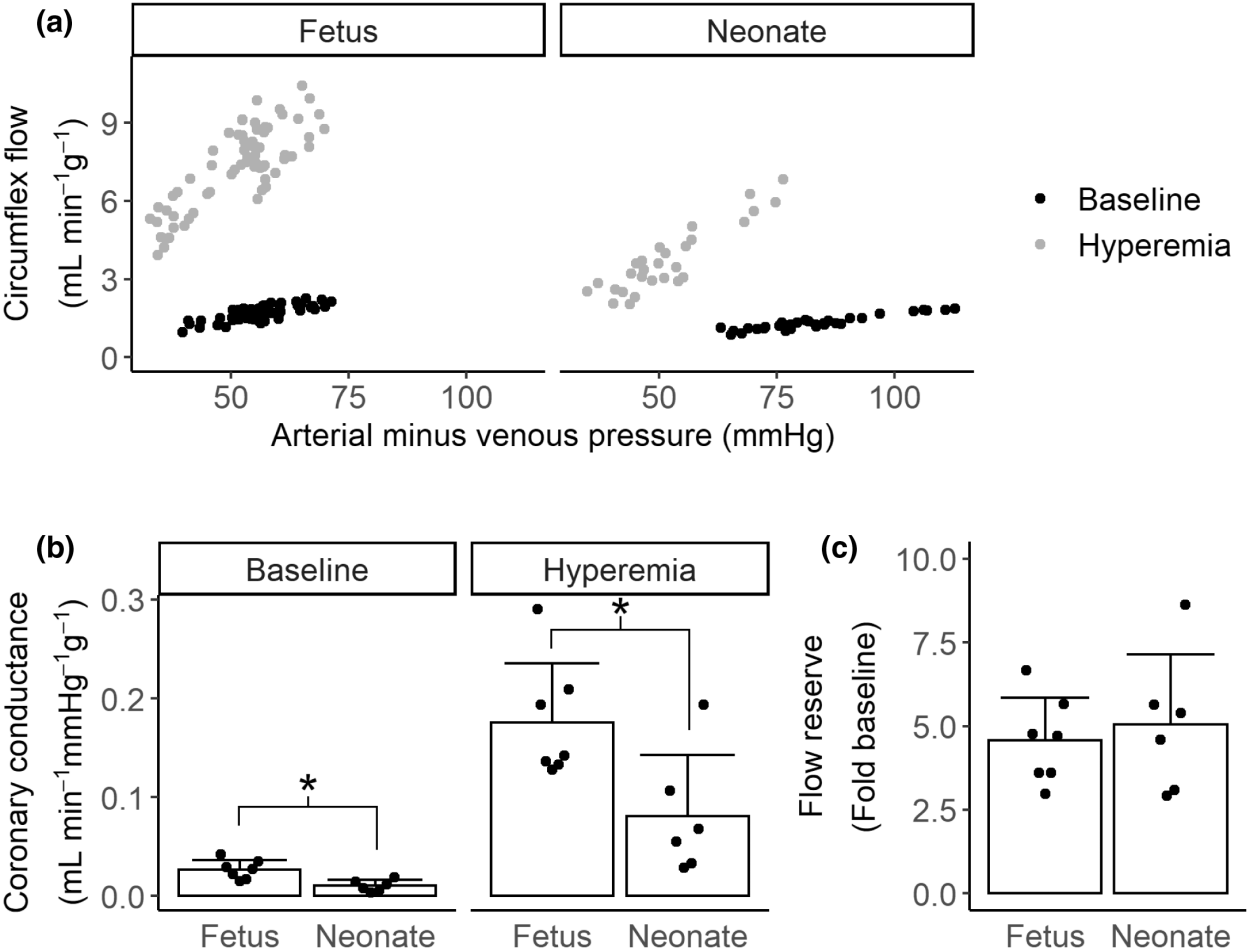
Pressure flow relationships assessed in fetuses (135 or 136 dGA, *N* = 7) and lambs (5 or 6 dPNA, *N* = 6). (a) Coronary pressure‐flow relationship examples from a single fetus (left) and a single neonate (right). Black points show baseline data and gray points show hyperemic data. Each point represents pressure‐flow data averaged over 1 s. (b) Coronary conductance at baseline (left panel) was lower in the neonate than the fetus (*t*‐test, *p* = 0.003), as it was with adenosine hyperemia (right panel, *t*‐test, *p* = 0.018). (c) Flow reserve was not significantly different between fetuses and neonates (*t*‐test *p* = 0.641). Columns are means, error bars + *SD*, dots are individual data. *p < 0.05.

Example adenosine dose–response curves are shown in Figure S1. The minimum dose within the plateau phase of the adenosine dose–response relationship, determined the day before the pressure flow relationship, was not different (Figure S1, *p* = 0.993) between neonates (160 ± 92 μg kg^−1^ min^−1^) and fetuses (159 ± 47 μg kg^−1^ min^−1^). Example pressure‐flow relationships from a fetus and from a lamb are shown in Figure 3a. Coronary conductance at baseline and with adenosine hyperemia was significantly lower in neonates than fetuses (Figure 3b, baseline 1.02 × 10^−2^ ± 5.9 × 10^−3^ vs 2.68 × 10^−2^ ± 9.6 × 10^−3^ ml min^−1^ mmHg^−1^ g^−1^, *p* = 0.003; adenosine hyperemia 8.07 × 10^−2^ ± 6.2 × 10^−2^ vs 1.76 × 10^−1^ ± 6.0 × 10^−2^ ml min^−1^ mmHg^−1^ g^−1^, *p* = 0.018). Flow reserve was not different between the age groups (Figure 3c, neonate 5.04 ± 2.09 vs fetus 4.56 ± 1.29, *p* = 0.642).

### 3.4 Microvascular blood flow (anesthetized)

Blood contents and hemodynamics measured immediately before MCE studies (under the influence of maternal anesthesia (fetuses), anesthesia (neonates), atropine, and propranolol) are reported in Table 3. Anesthetized neonates and fetuses were acidemic compared to unanesthetized (Table 2), with fetal pH being lower than neonatal. Anesthetized neonatal PO_2_ was an order of magnitude greater than fetal, and neonatal oxygen content is about 90% higher than fetal. In contrast to the unanesthetized situation, arterial minus venous pressure and heart rate are not significantly different between fetuses and neonates and the RPP in the neonate is about half of that in the fetus.

**TABLE 3:** Anesthetized arterial pH, oxygen content, and hemodynamics

	Fetus	Neonate	*p*‐value
pH	7.194 ± 0.019	7.280 ± 0.039	0.044
PO_2_	22 ± 5	467 ± 13	<0.0001
O_2_ content (mg/dl)	6.7 ± 3.0	12.7 ± 1.6	0.021
Arterial—venous pressure (mmHg)	49.7 ± 9.5	42.0 ± 8.6	NS
Heart rate (min^−1^)	151 ± 13	109 ± 53	NS
RPP (mmHg min^−1^)	7211 ± 1774	3796 ± 1442	0.038

*Note*: Measurements made just prior to MCE studies in exteriorized fetuses with maternal anesthesia (135 or 136 dGA, *N* = 4) or in neonates under anesthesia (5 dPNA, *N* = 3). Recordings made following autonomic blockade with atropine and propranolol. RPP (rate pressure product). Mean ± *SD*. Data analyzed by *t*‐test. *p* > 0.05 considered not statistically significant (NS).

**FIGURE 4:**
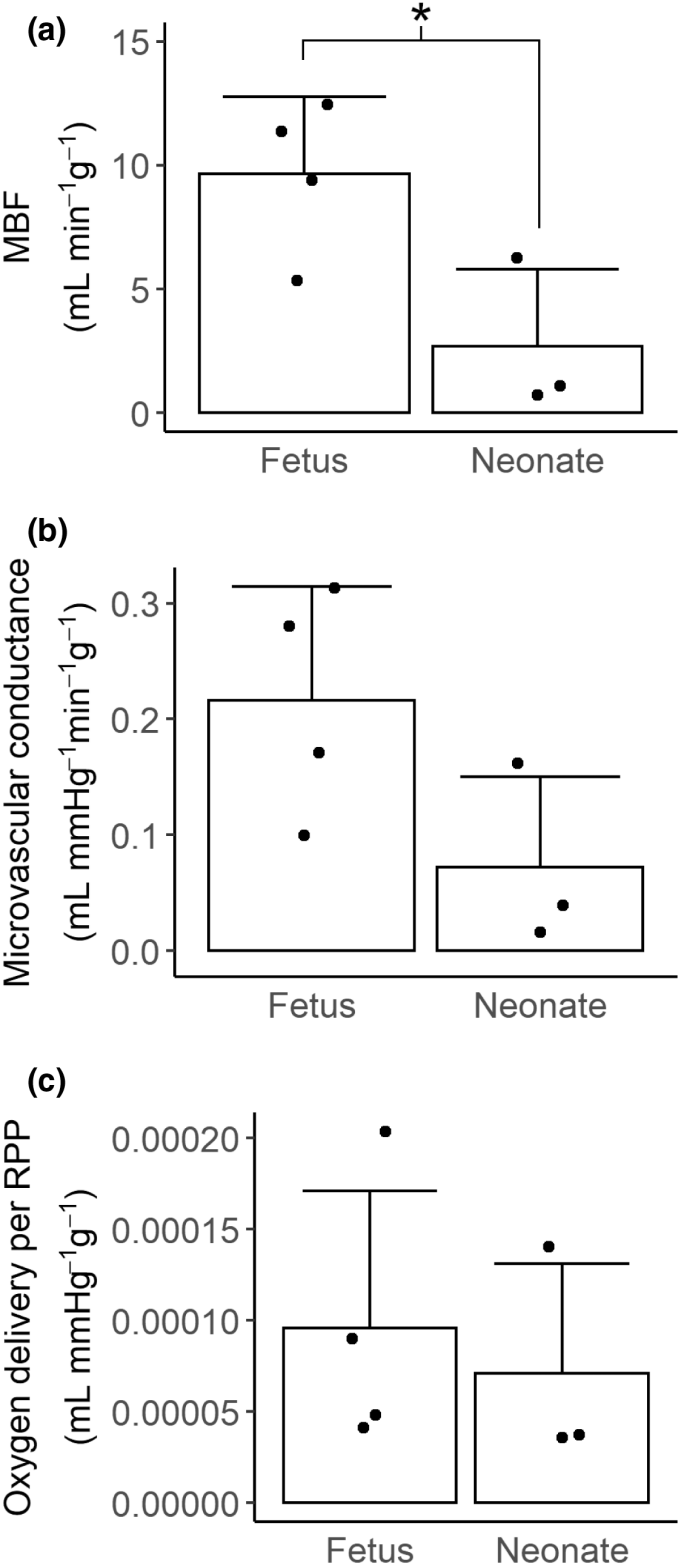
Microvascular coronary perfusion. A subset of fetuses (135 or 136 dGA, *N* = 4) and lambs (5 dPNA, *N* = 3) in the flow probe cohorts had microvascular perfusion assessed by MCE. (a) MBF was significantly lower in neonates than fetuses (*t*‐test, *p* = 0.037). (b) Microvascular conductance trended lower in the neonate relative to the fetus (*t*‐test, *p* = 0.085). (c) Oxygen delivery per RPP per gram of myocardium as measured by MCE was not significantly different between fetuses and neonates (*t*‐test, *p* = 0.651). Columns are means, error bars + *SD*, dots are individual data. *p < 0.05.

MBF was significantly lower in neonates than fetuses (Figure 4a, 2.69 ± 3.10 vs 9.64 ± 3.13 ml min^−1^ g^−1^, *p* = 0.037). Microvascular conductance was similarly lower in neonates compared to fetuses, but did not reach statistical significance (Figure 4b, 0.072 ± 0.078 vs 0.216 ± 0.098 ml mmHg^−1^ min^−1^ g^−1^, *p* = 0.085). Oxygen delivery per RPP was not significantly different between age groups (Figure 4c, neonatal 7.12 × 10^−5^ ± 6.0 × 10^−5^ vs fetal 9.58 × 10^−5^ ± 7.5 × 10^−5^ ml mmHg^−1^ g^−1^, *p* = 0.651).

### 3.5 Oxygen extraction (unanesthetized)

**FIGURE 5:**
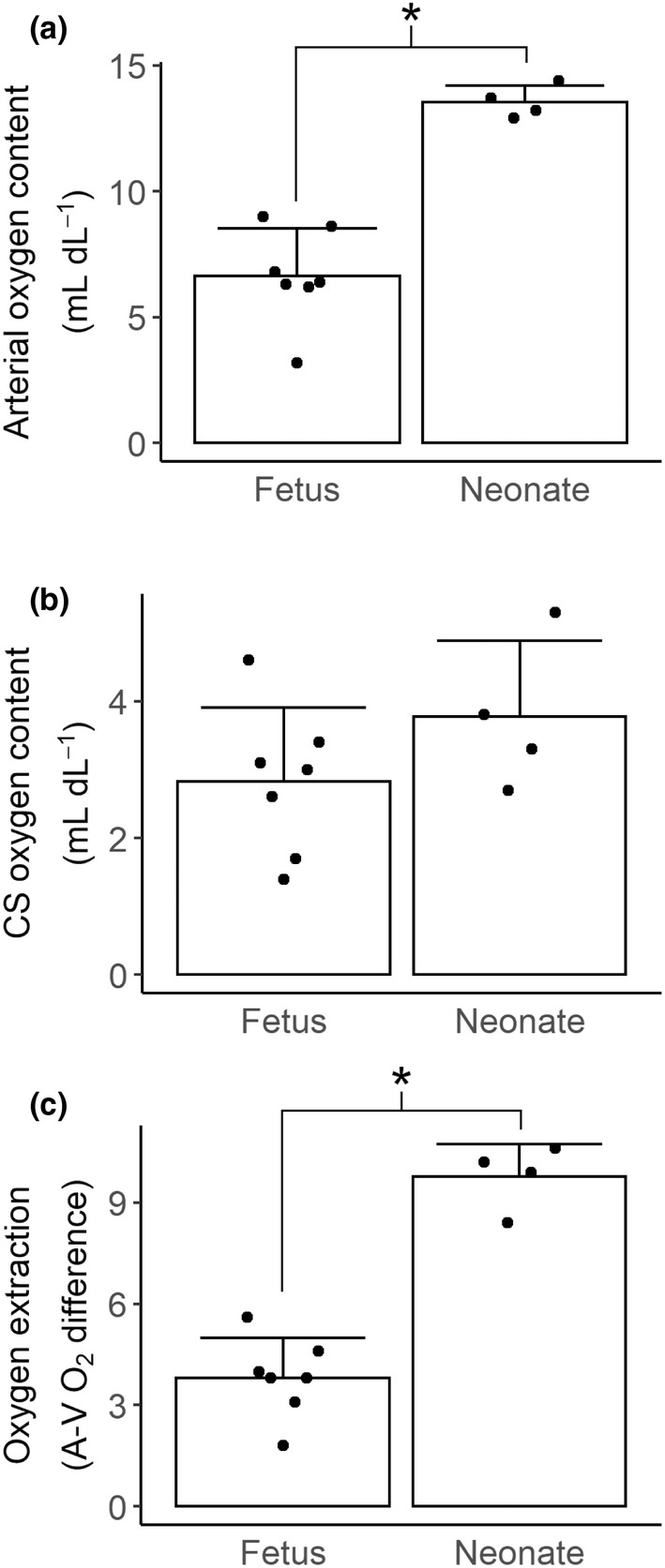
Oxygen extraction by the myocardium. Cohorts of fetuses (134 dGA, *N* = 7) and lambs (5 dPNA, *N* = 4), separate to those in which flow was measured, had simultaneous samples of arterial and coronary sinus blood sampled for the determination of oxygen extraction by the myocardium. (a) Arterial oxygen content was significantly higher in neonates than fetuses (*t*‐test, *p* < 0.001). (b) Coronary sinus oxygen content was not significantly different between fetuses and neonates (*t*‐test, *p* = 0.217). (c) Oxygen extraction by the neonatal myocardium was significantly greater than the fetal myocardium (*t*‐test, *p* < 0.001). Columns are means, error bars + *SD*, dots are individual data. *p < 0.05.

In the coronary sinus cohorts, neonatal arterial oxygen content was more than double fetal arterial oxygen content (Figure 5a, 6.6 ± 1.9 vs 13.6 ± 0.7 ml dl^−1^, *p* < 0.001), and coronary sinus oxygen content was not different between fetuses and neonates (Figure 5b, 3.8 ± 1.1 vs 2.8 ± 1.1 ml dl^−1^, *p* = 0.217). Thus, oxygen extraction, the difference between arterial and venous oxygen contents, was significantly greater in neonates than fetuses (Figure 5c, 9.8 ± 0.9 vs 3.8 ± 1.2 ml dl^−1^, *p* < 0.001).

### 3.6 Extrapolated oxygen consumption (unanesthetized)

**FIGURE 6:**
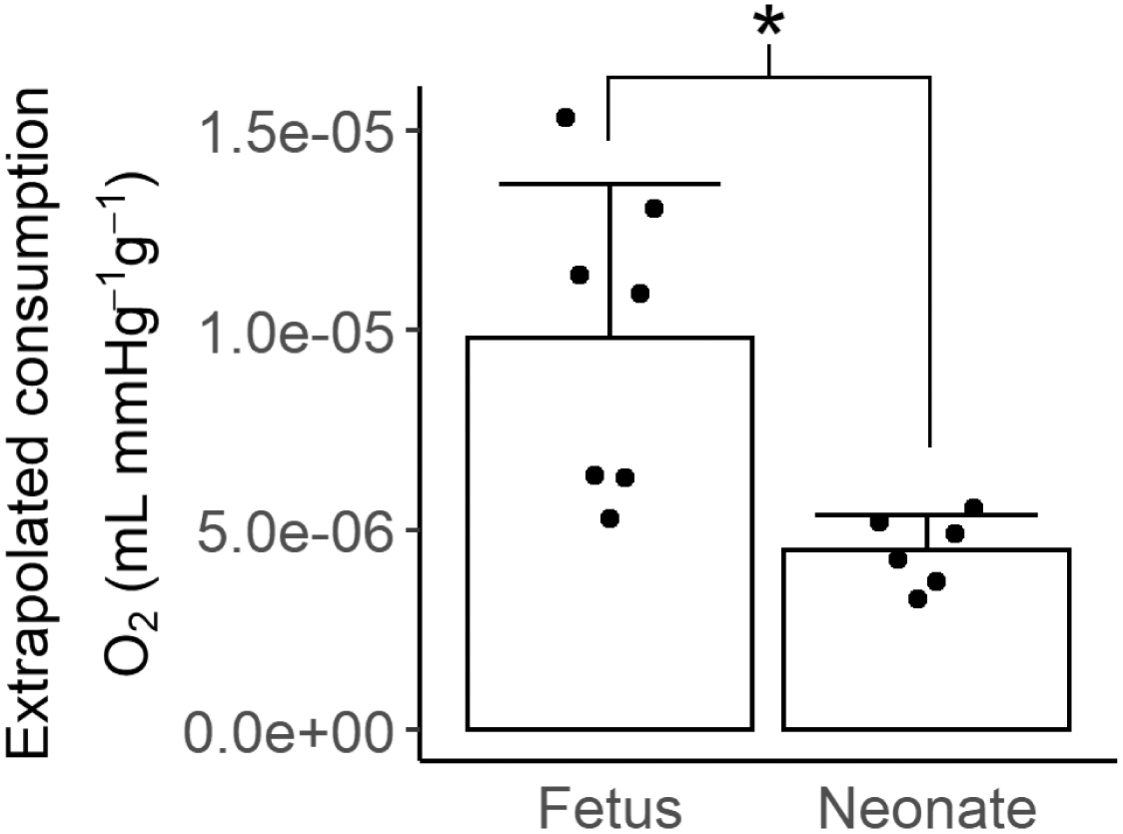
Extrapolated oxygen consumption by the myocardium. Oxygen consumption was estimated by applying the mean coronary sinus oxygen content values obtained in the oxygen extraction cohort (*N* = 7 fetuses, 4 lambs) to the coronary flow cohort (*N* = 7 fetuses, 6 lambs). The extrapolated oxygen consumption per RPP was significantly lower in lambs than fetuses (Mann–Whitney *U*‐test, *p* = 0.002). Columns are means, error bars + *SD*, dots are individual data. *p < 0.05.

Oxygen consumption was extrapolated from coronary sinus oxygen content from the coronary sinus cohorts, and arterial oxygen content and coronary flow from the coronary flow cohorts. Extrapolated oxygen consumption per RPP was less than half in the neonate what it was in the fetus (Figure 6; 4.5 × 10^−6^ vs 9.8 × 10^−6^ ml min^−1^ mmHg^−1^ g^−1^, *p* = 0.002).

## 4 DISCUSSION

### 4.1 Perinatal physiology

The central physiological finding of this study is that, relative to the late gestation fetus, the myocardium of the newborn has less perfusion, less capacity for perfusion, and receives and consumes less oxygen at rest. These changes occur in the context of complex changes in cardiovascular physiology at birth. As systemic vascular resistance sharply increases, the fetal shunts close, and systemic metabolic demand increases (Morton & Brodsky, 2016), consequently the rate pressure product doubles (Table 2). Concurrently, arterial oxygen content increases with ventilation. We discuss these results in the context of earlier studies of coronary vascular function over developmental time and speculate on the mechanisms driving them. Two primary drivers of lower myocardial perfusion in the neonate relative to the late gestation fetus may be more rapid myocyte growth than capillary growth, and metabolic demand due to myocyte proliferation and terminal differentiation which is not captured by RPP.

### 4.2 Previous studies of perinatal changes in coronary vascular function

Few studies to date have characterized myocardial perfusion in the immediate perinatal period. Dalshaug and colleagues used radioactive microspheres to show that weight‐normalized resting perfusion of both the RV and LV is lower at 128 dGA than 100 dGA, suggesting that resting flow decreases with increasing gestational age (Dalshaug et al., 2002). In contrast, Fisher and colleagues used radioactive microspheres to measure resting myocardial blood flow and oxygen delivery in fetal (123 dGA), neonatal (4–23 dPNA), and adult sheep (Fisher et al., 1982); relative to fetuses, neonates had lower RV perfusion, unchanged septal perfusion, and higher LV perfusion. While our use of the Evan's blue procedure to determine distribution area of measured flow does not allow us to measure flow distribution as the microsphere method does, we know that flow through the circumflex predominantly measures perfusion of the left heart and interventricular septum, and only small portions of the RV (Figure 2a). Thus, our results contrast with Fisher's. Like us, Fisher measured flow in unanesthetized animals following surgical instrumentation, meaning that the source of the discordance in results is most likely not a difference in methodologies, but rather a result of the differences in precise ages studied. Prenatally, the RV and LV grow at similar rates, but postnatally LV hypertrophic growth quickly outpaces that of the RV (Jonker et al., 2015). Putting our data in the context of Dalshaug's and Fisher's, we postulate that resting left ventricular perfusion decreases through mid‐ and late gestation and parturition, before transiently increasing during the first weeks of postnatal life. Another possibility for the different findings between us and Fisher is in the precise cardiac location studied; we might have seen increased resting neonatal perfusion had we instrumented the left anterior descending rather than the circumflex artery. However, that our MCE results also showed lower neonatal than fetal perfusion of the anterior LV free wall (albeit under anesthesia) make this unlikely.

### 4.3 Multiple methods for measuring myocardial perfusion

A secondary goal of this study was to assess the utility of MCE as a tool to define normal developmental changes in myocardial microvascular perfusion. MCE is advantageous based on its minimal invasiveness, capability for anatomical targeting, and ability to separate flow into the components of fractional volume (indicating capillary density) and flux rate (indicating capillary diameter or blood viscosity) (Kaufmann et al., 2007; Lindner, 2021). However, MCE in experimental animals is usually carried out under anesthesia. We have previously used MCE to expand our understanding of coronary microvascular adaptations to anemia in fetal sheep (Jonker et al., 2016). In this MCE pilot study, complementary to our study with transit time flow probes, we found similar trends in resting perfusion, conductance, and oxygen delivery (Figure 4). Given our very limited pilot sample sizes, we do not present fractional volume or flux rate measurements to avoid making claims which we cannot statistically substantiate.

Our use of general anesthesia in the fetuses was deemed necessary to partially exteriorize and access the fetal chest, and was also used in the neonate for consistency. However, isoflurane is a coronary and peripheral vasodilator (Priebe, 1987), which limits our ability to compare the flow probe and MCE datasets one‐to‐one. This may also partially explain the higher flows seen in the MCE relative to flow probe datasets. To our knowledge, MCE data in fetal and neonatal sheep have not been previously reported in real units; our earlier study used uncorrected video intensity measurements instead (Jonker et al., 2016). Blood pool normalization depends on assumptions of steady state contrast agent infusion and clearance which have been demonstrated across animal models (Kaufmann et al., 2007), but have not been previously characterized in fetal or neonatal sheep. In spite of these limitations, the broadly similar trends seen between these methods is encouraging for future applications of MCE to more in‐depth study of normal development.

### 4.4 Explanations for decreased neonatal coronary perfusion

#### 4.4.1 Oxygen metabolism

Given that a key function of arterial blood flow is oxygen delivery, the most obvious explanation for the lower resting flow in the neonate relative to fetus is coronary autoregulation due to the higher arterial oxygen content in postnatal life. Indeed, oxygen delivery per time was equivalent between fetuses and neonates despite lower circumflex flow in the neonate. However, the reduction in neonatal oxygen delivery normalized to RPP compared to the fetus was an unexpected finding (Figure 2c). We added an additional cohort to investigate whether these differences were a result of increased blood oxygen extraction in the neonate. We did find significantly greater oxygen extraction in the neonate than the fetus (Figure 5). However, when we applied the mean coronary sinus oxygen contents from the oxygen extraction cohort to the oxygen delivery values obtained in the coronary flow cohort to calculate extrapolated oxygen consumption per RPP (Figure 6), we found significantly greater fetal than neonatal oxygen consumption. While this may be due to changes in the efficiency of oxygen extraction, changes in the oxygen requirement for cardiac function, or metabolic needs separate to cardiac work, we believe that our results are driven by the latter mechanism, as discussed below.

It is possible that the metabolic need for oxygen in the fetal myocardium is simply higher than that of the neonatal myocardium. With the initiation of feeding after birth, fatty acids are added to the energy sources powering cardiomyocytes (Bartelds et al., 2000; Morton & Brodsky, 2016), however fatty acid metabolism requires more oxygen per high‐energy phosphate bond produced than glucose metabolism. Instead, the fetal myocardium may require energy for work not captured by the RPP. The process of cell division is thermally demanding (Rodenfels et al., 2019), as is the process of the DNA replication required for even non‐mitotic nuclear replication leading to binculeation (Lynch & Marinov, 2015), suggesting that the extra work performed by the fetal myocardium could be that of proliferation and terminal differentiation. This notion is further supported by the outsized energy demands of cell proliferation seen in cancer (Friesen et al., 2015). This may explain the observed mismatch between RPP and oxygen consumption.

#### 4.4.2 Myocardial growth

The most likely explanation for our finding of lower neonatal relative to fetal capacity for myocardial perfusion is that cardiac myofibers are growing more quickly than the vascular system which supports them. Heart weight roughly doubled in mass during the 17 days separating our fetal and neonatal time points (Table 1), however absolute flow through the circumflex artery was not different between age groups (Table 2). Growth patterns of cardiac myocytes are well defined during this period of transition from hyperplastic to hypertrophic growth in both sheep and humans (Austin et al., 1995; Burrell et al., 2003; Jonker et al., 2015). Fetuses at 135 dGA are in the midst of a prenatal apoptotic wave of cardiomyocyte attrition, simultaneous to a substantial proportion of the cardiomyocyte population terminally differentiating (Jonker et al., 2015). Less clearly defined is what is happening to coronary vascular development at this time. Lambs are able to maintain coronary vascular reserve in the face of hypertrophy caused by aortic banding in a way that adult sheep are not (Flanagan et al., 1991, 1994). The timing of that loss of plasticity is unclear, but may be a gradual process beginning at birth. Ultimately our finding that maximal flow capacity per gram of myocardium is reduced after birth is a functional correlate to histological findings which show that the number of capillaries per cardiac myofiber decreases gradually until adulthood (Roberts & Wearn, 1941; Shipley et al., 1937; Smolich et al., 1989).

#### 4.4.3 Microvascular contribution to coronary resistance

While classical ideas about the regulation of resistance in the coronary system focus on arterioles, evidence in recent decades has pointed to the centrality of the capillary microvasculature. It has been found that coronary capillary recruitment is involved in flow responses to increased demand and experimentally induced hyperemia and stenosis in adult dogs (Jayaweera et al., 1999; Le et al., 2002). Indeed, capillary recruitment is a mechanism for increasing coronary flow in the fetal heart (Jonker et al., 2016). However, if derecruitment were the mechanism by which neonatal coronary flow was less, coronary reserve would be expected to increase in the neonate, which it did not.

When considering the contribution of capillaries to coronary vascular resistance, the particular rheology of these very small vessels should be considered. We observed the decline in hematocrit typical of the newborn (Jopling et al., 2009), which decreases blood viscosity. This is an important contributor to coronary flow dynamics (Pries et al., 1994), as underscored by studies of fetal anemia demonstrating that increases in hyperemic flow in anemic animals are partially reversible when restoring normal hematocrit (Davis et al., 1999), and MCE studies showing that increases in anemic flow are attributable to increased flux rate not fractional volume (Jonker et al., 2016). This change in hematocrit would tend to increase coronary flow, and consequently cannot be an explanation for the reduced coronary flow in the neonate.

#### 4.4.4 Coronary flow reserve

We found no significant change in coronary flow reserve between the late gestation fetus and early neonate. This was unexpected for two reasons; first our understanding of the increased systemic demands of postnatal life (Morton & Brodsky, 2016) led us to hypothesize that reserve would necessarily increase postnatally, and second, earlier studies of coronary vascular function during gestation showed an increase in reserve between 100 and 128 dGA (Dalshaug et al., 2002). Notably our reserve data were highly variable, with most animals falling between reserve values of 2.5 and 6 (Figure 3). This variation is reflected in both earlier reports of fetal lamb reserve around this point in gestation (Jonker et al., 2020) and in clinical data showing that healthy human juvenile and adult coronary flow reserve ranges between 2 and 4 fold (Lim et al., 2000; Óskarsson, 2007). Overall, preservation of similar coronary reserve between the fetus and neonate suggests that vascular anatomy relative to cardiomyocyte volume (i.e., flow capacity) changes in the days surrounding birth, but that basal coronary tone does not.

### 4.5 Study limitations

This technically challenging study could only be completed by use of a large animal model. Our choice of methodologies results in tradeoffs and inevitable study limitations which must be acknowledged. First, the Evan's blue procedure enables an accurate mass normalization of our circumflex flow data. However, because of potential differences in mass specific flow and microvascular reactivity, it does not allow us to accurately determine the distribution of circumflex flow to the heart structures we studied, as the microsphere method does (Prinzen & Bassingthwaighte, 2000), and as the MCE method does. Second, the use of anesthesia during the MCE pilot studies, necessary for reliable fetal imaging, results in a sufficiently different hemodynamic environment that our micro‐ and macrovascular flow data cannot be compared one‐to‐one. Finally, our use of separate flow probe and coronary sinus cohorts prevents us from being able to directly measure oxygen consumption.

## 5 CONCLUSION

We have identified that there are reductions immediately following birth in resting coronary flow, maximal flow capacity, and oxygen delivery to and consumption by the myocardium downstream of the proximal circumflex artery. We found that coronary flow reserve was preserved but not increased between the fetal and newborn periods. These changes occur in the context of rapid myocardial growth, changes to blood viscosity due to decreasing hematocrit, and a changing metabolic landscape. The differences we found between the late gestation fetal and newborn hearts are likely functional manifestations of previously defined broad anatomical trends toward reduced capillary density approaching maturity.

## AUTHOR CONTRIBUTIONS

SSJ conceived the study. MWH, SL, SMA, JRL and SSJ designed the methodology. MWH, SL, SMA and SSJ conducted the research and collected the data. MWH analyzed the data and prepared the original draft. All authors participated in interpretation of the results, in review and editing, and approved the final draft.

## Supporting information


Figure S1.

## References

[phy215523-bib-0001] Adler, C. P. , & Costabel, U. (1975). Cell number in human heart in atrophy, hypertrophy, and under the influence of cytostatics. Recent Advances in Studies on Cardiac Structure and Metabolism, 6, 343–355. 128080

[phy215523-bib-0002] Austin, A. , Fagan, D. G. , & Mayhew, T. M. (1995). A stereological method for estimating the total number of ventricular myocyte nuclei in fetal and postnatal hearts. Journal of Anatomy, 187(Pt 3), 641–647. 8586563 PMC1167467

[phy215523-bib-0003] Bartelds, B. , Knoester, H. , Smid, G. B. , Takens, J. , Visser, G. H. , Penninga, L. , van der Leij, F. R. , Beaufort‐Krol, G. C. M. , Zijlstra, W. G. , Heymans, H. S. A. , & Kuipers, J. R. G. (2000). Perinatal changes in myocardial metabolism in lambs. Circulation, 102, 926–931. 10952964 10.1161/01.cir.102.8.926

[phy215523-bib-0004] Burrell, J. H. , Boyn, A. M. , Kumarasamy, V. , Hsieh, A. , Head, S. I. , & Lumbers, E. R. (2003). Growth and maturation of cardiac myocytes in fetal sheep in the second half of gestation. The Anatomical Record Part A, Discoveries in Molecular, Cellular, and Evolutionary Biology, 274, 952–961. 12973719 10.1002/ar.a.10110

[phy215523-bib-0005] Dalshaug, G. B. , Scholz, T. D. , Smith, O. M. , Bedell, K. A. , Caldarone, C. A. , & Segar, J. L. (2002). Effects of gestational age on myocardial blood flow and coronary flow reserve in pressure‐loaded ovine fetal hearts. American Journal of Physiology‐Heart and Circulatory Physiology, 282, H1359–H1369. 11893572 10.1152/ajpheart.00686.2001

[phy215523-bib-0006] Davis, L. , Roullet, J. B. , Thornburg, K. L. , Shokry, M. , Hohimer, A. R. , & Giraud, G. D. (2003). Augmentation of coronary conductance in adult sheep made anaemic during fetal life. The Journal of Physiology, 547, 53–59. 12562949 10.1113/jphysiol.2002.023283 PMC2342629

[phy215523-bib-0007] Davis, L. E. , Hohimer, A. R. , & Morton, M. J. (1999). Myocardial blood flow and coronary reserve in chronically anemic fetal lambs. American Journal of Physiology‐Regulatory, Integrative and Comparative Physiology, 277, R306–R313. 10.1152/ajpregu.1999.277.1.R306 10409287

[phy215523-bib-0008] Fisher, D. J. , Heymann, M. A. , & Rudolph, A. M. (1982). Regional myocardial blood flow and oxygen delivery in fetal, newborn, and adult sheep. American Journal of Physiology‐Heart and Circulatory Physiology, 243, H729–H731. 10.1152/ajpheart.1982.243.5.H729 6814267

[phy215523-bib-0009] Flanagan, M. F. , Aoyagi, T. , Currier, J. J. , Colan, S. P. , & Fujii, A. M. (1994). Effect of young age on coronary adaptations to left ventricular pressure overload hypertrophy in sheep. Journal of the American College of Cardiology, 24, 1786–1796. 7963129 10.1016/0735-1097(94)90188-0

[phy215523-bib-0010] Flanagan, M. F. , Fujii, A. M. , Colan, S. D. , Flanagan, R. G. , & Lock, J. E. (1991). Myocardial angiogenesis and coronary perfusion in left ventricular pressure‐overload hypertrophy in the young lamb. Evidence for inhibition with chronic protamine administration. Circulation Research, 68, 1458–1470. 1708312 10.1161/01.res.68.5.1458

[phy215523-bib-0011] Friesen, D. E. , Baracos, V. E. , & Tuszynski, J. A. (2015). Modeling the energetic cost of cancer as a result of altered energy metabolism: Implications for cachexia. Theoretical Biology and Medical Modelling, 12, 17. 26370269 10.1186/s12976-015-0015-0 PMC4570294

[phy215523-bib-0012] Giraud, G. D. , Faber, J. J. , Jonker, S. S. , Davis, L. , & Anderson, D. F. (2006). Effects of intravascular infusions of plasma on placental and systemic blood flow in fetal sheep. American Journal of Physiology Heart and Circulatory Physiology, 291, H2884–H2888. 16905601 10.1152/ajpheart.00428.2006

[phy215523-bib-0013] Jayaweera, A. R. , Wei, K. , Coggins, M. , Bin, J. P. , Goodman, C. , & Kaul, S. (1999). Role of capillaries in determining CBF reserve: New insights using myocardial contrast echocardiography. American Journal of Physiology‐Heart and Circulatory Physiology, 277, H2363–H2372. 10.1152/ajpheart.1999.277.6.H2363 10600857

[phy215523-bib-0014] Jonker, S. S. , Davis, L. E. , Soman, D. , Belcik, J. T. , Davidson, B. P. , Atkinson, T. M. , Wilburn, A. , Louey, S. , Giraud, G. D. , & Lindner, J. R. (2016). Functional adaptations of the coronary microcirculation to anaemia in fetal sheep. The Journal of Physiology, 594, 6165–6174. 27291778 10.1113/JP272696 PMC5088228

[phy215523-bib-0015] Jonker, S. S. , Giraud, G. D. , Chang, E. I. , Elman, M. R. , & Louey, S. (2020). Coronary vascular growth matches IGF‐1‐stimulated cardiac growth in fetal sheep. FASEB Journal, 34, 10041–10055. 32573852 10.1096/fj.202000215R PMC7688557

[phy215523-bib-0016] Jonker, S. S. , Louey, S. , Giraud, G. D. , Thornburg, K. L. , & Faber, J. J. (2015). Timing of cardiomyocyte growth, maturation, and attrition in perinatal sheep. FASEB Journal, 29, 4346–4357. 26139099 10.1096/fj.15-272013 PMC4566940

[phy215523-bib-0017] Jopling, J. , Henry, E. , Wiedmeier, S. E. , & Christensen, R. D. (2009). Reference ranges for hematocrit and blood hemoglobin concentration during the neonatal period: Data from a multihospital health care system. Pediatrics, 123, e333–e337. 19171584 10.1542/peds.2008-2654

[phy215523-bib-0018] Karamlou, T. , Giraud, G. D. , McKeogh, D. , Jonker, S. S. , Shen, I. , Ungerleider, R. M. , & Thornburg, K. L. (2019). Right ventricular remodeling in response to volume overload in fetal sheep. American Journal of Physiology‐Heart and Circulatory Physiology, 316, H985–H991. 30707615 10.1152/ajpheart.00439.2018 PMC6580393

[phy215523-bib-0019] Kaufmann, B. A. , Wei, K. , & Lindner, J. R. (2007). Contrast echocardiography. Current Problems in Cardiology, 32, 51–96. 17208647 10.1016/j.cpcardiol.2006.10.004

[phy215523-bib-0020] Le, D. E. , Bin, J.‐P. , Coggins, M. P. , Wei, K. , Lindner, J. R. , & Kaul, S. (2002). Relation between myocardial oxygen consumption and myocardial blood volume: A study using myocardial contrast echocardiography. Journal of the American Society of Echocardiography, 15, 857–863. 12221400 10.1067/mje.2002.121275

[phy215523-bib-0021] Lim, H. E. , Shim, W. J. , Rhee, H. , Kim, S. M. , Hwang, G. S. , Kim, Y. H. , Seo, H. S. , Oh, D. J. , & Ro, Y. M. (2000). Assessment of coronary flow reserve with transthoracic doppler echocardiography: Comparison among adenosine, standard‐dose dipyridamole, and high‐dose dipyridamole. Journal of the American Society of Echocardiography, 13, 264–270. 10756243 10.1067/mje.2000.103508

[phy215523-bib-0022] Lindner, J. R. (2021). Contrast echocardiography: Current status and future directions. Heart, 107, 18–24. 33077502 10.1136/heartjnl-2020-316662

[phy215523-bib-0023] Lu, P. , Wang, Y. , Liu, Y. , Wang, Y. , Wu, B. , Zheng, D. , Harvey, R. P. , & Zhou, B. (2021). Perinatal angiogenesis from pre‐existing coronary vessels via DLL4–NOTCH1 signalling. Nature Cell Biology, 23, 967–977. 34497373 10.1038/s41556-021-00747-1

[phy215523-bib-0024] Lynch, M. , & Marinov, G. K. (2015). The bioenergetic costs of a gene. Proceedings of the National Academy of Sciences, 112, 15690–15695. 10.1073/pnas.1514974112 PMC4697398 26575626

[phy215523-bib-0025] Morton, S. U. , & Brodsky, D. (2016). Fetal physiology and the transition to extrauterine life. Clinics in Perinatology, 43, 395–407. 27524443 10.1016/j.clp.2016.04.001 PMC4987541

[phy215523-bib-0026] Óskarsson, G. (2007). Coronary flow and flow reserve in children. Acta Paediatrica, 93, 20–25. 10.1080/08035320410022749 15702666

[phy215523-bib-0027] Priebe, H. J. (1987). Differential effects of isoflurane on regional right and left ventricular performances, and on coronary, systemic, and pulmonary hemodynamics in the dog. Anesthesiology, 66, 262–272. 3826683 10.1097/00000542-198703000-00002

[phy215523-bib-0028] Pries, A. R. , Secomb, T. W. , Gessner, T. , Sperandio, M. B. , Gross, J. F. , & Gaehtgens, P. (1994). Resistance to blood flow in microvessels in vivo. Circulation Research, 75, 904–915. 7923637 10.1161/01.res.75.5.904

[phy215523-bib-0029] Prinzen, F. W. , & Bassingthwaighte, J. B. (2000). Blood flow distributions by microsphere deposition methods. Cardiovascular Research, 45, 13–21. 10728307 10.1016/s0008-6363(99)00252-7 PMC3483311

[phy215523-bib-0030] R Core Team (2022). R: a language and environment for statistical computing. Available at: https://www.r‐project.org/.

[phy215523-bib-0031] Roberts, J. T. , & Wearn, J. T. (1941). Quantitative changes in the capillary‐muscle relationship in human hearts during normal growth and hypertrophy. American Heart Journal, 21, 617–633.

[phy215523-bib-0032] Rodenfels, J. , Neugebauer, K. M. , & Howard, J. (2019). Heat oscillations driven by the embryonic cell cycle reveal the energetic costs of signaling. Developmental Cell, 48, 646–658.e6. 30713074 10.1016/j.devcel.2018.12.024 PMC6414255

[phy215523-bib-0033] Shipley, R. A. , Shipley, L. J. , & Wearn, J. T. (1937). The capillary supply in normal and hypertrophied hearts of rabbits. Journal of Experimental Medicine, 65, 29–42. 19870589 10.1084/jem.65.1.29 PMC2133467

[phy215523-bib-0034] Singer, D. , & Mühlfeld, C. (2007). Perinatal adaptation in mammals: The impact of metabolic rate. Comparative Biochemistry and Physiology Part A: Molecular & Integrative Physiology, 148, 780–784. 10.1016/j.cbpa.2007.05.004 17561425

[phy215523-bib-0035] Smolich, J. J. , Walker, A. M. , Campbell, G. R. , & Adamson, T. M. (1989). Left and right ventricular myocardial morphometry in fetal, neonatal, and adult sheep. American Journal of Physiology‐Heart and Circulatory Physiology, 257, H1–H9. 10.1152/ajpheart.1989.257.1.H1 2750930

[phy215523-bib-0036] Tian, X. , Hu, T. , Zhang, H. , He, L. , Huang, X. , Liu, Q. , Yu, W. , He, L. , Yang, Z. , Yan, Y. , Yang, X. , Zhong, T. P. , Pu, W. T. , & Zhou, B. (2014). Vessel formation. De novo formation of a distinct coronary vascular population in neonatal heart. Science, 345, 90–94. 24994653 10.1126/science.1251487 PMC4275002

